# A Temperate *Sinorhizobium* Phage, AP-16-3, Closely Related to Phage 16-3: Mosaic Genome and Prophage Analysis

**DOI:** 10.3390/v15081701

**Published:** 2023-08-06

**Authors:** Alexandra P. Kozlova, Alla S. Saksaganskaia, Alexey M. Afonin, Victoria S. Muntyan, Maria E. Vladimirova, Elena A. Dzyubenko, Marina L. Roumiantseva

**Affiliations:** 1 Laboratory of Genetics and Selection of Microorganisms, Federal State Budget Scientific Institution All-Russia Research Institute for Agricultural Microbiology (FSBSI ARRIAM), 196608 Saint Petersburg, Russia; a.kozlova@arriam.ru (A.P.K.); allasaksaganskaya@arriam.ru (A.S.S.); vucovar@yandex.ru (V.S.M.); mariiacherkasova@arriam.ru (M.E.V.); 2 Laboratory of Genetics of Plant-Microbe Interactions, Federal State Budget Scientific Institution All-Russia Research Institute for Agricultural Microbiology (FSBSI ARRIAM), 196608 Saint Petersburg, Russia; afoninalexeym@gmail.com; 3 N. I. Vavilov Institute of Plant Genetic Resources (VIR), 190031 Saint Petersburg, Russia; elena.dzyubenko@gmail.com

**Keywords:** rhizobiophages, temperate tailed phages, *Sinorhizobium* spp., prophages, intergenomic similarity, dissolved phage genome, Vavilov’s centers of origin

## Abstract

Soil *Sinorhizobium* phage AP-16-3, a strain phylogenetically close to *Rhizobium* phage 16-3, was isolated in a mountainous region of Dagestan, belonging to the origin of cultivated plants in the Caucasus, according to Vavilov N.I. The genome of phage AP-16-3 is 61 kbp in size and contains 62 ORFs, of which 42 ORFs have homologues in the genome of *Rhizobium* phage 16-3, which was studied in the 1960s–1980s. A search for *Rhizobium* phage 16-3-related sequences was performed in the genomes of modern strains of root nodule bacteria belonging to different species, genera, and families. A total of 43 prophages of interest were identified out of 437 prophages found in the genomes of 42 strains, of which 31 belonged to *Sinorhizobium meliloti* species. However, almost all of the mentioned prophages contained single ORFs, and only two prophages contained 51 and 39 ORFs homologous to phages related to 16-3. These prophages were detected in *S. meliloti* NV1.1.1 and *Rh. leguminosarum* OyaliB strains belonging to different genera; however, the similarity level of these two prophages did not exceed 14.7%. Analysis of the orphan genes in these prophages showed that they encoded predominantly virion structural elements, but also enzymes and an extensive group of hypothetical proteins belonging to the L, S, and E regions of viral genes of phage 16-3. The data obtained indicate that temperate phages related to 16-3 had high infectivity against nodule bacteria and participated in intragenomic recombination events involving other phages, and in horizontal gene transfer between rhizobia of different genera. According to the data obtained, it is assumed that the repetitive lysogenic cycle of temperate bacteriophages promotes the dissolution of the phage genetic material in the host bacterial genome, and radical updating of phage and host bacterial genomes takes place.

## 1. Introduction

Soil viruses are more numerous and diverse than viruses from other ecosystems [1] due to the wide variety of available agroecological niches and potential biological hosts. Viruses are an essential component of the soil microbiome and have a significant impact on bacterial population density and diversity; in turn, they have an effect on the composition of the microbial community [2,3,4,5]. It has been calculated that phages infect more than 10^23^ bacteria per second, making their role as agents for horizontal gene transfer between bacterial hosts of the same or different species more plausible [6]. Bacteriophages can play a significant role as reservoirs of variable foreign sequences accumulated due to their infection activity. Bacteriophages have been reported to host different antibiotic resistance genes [7]. Many phage enzymes have novel properties, and some of which have important applications in molecular biology and biotechnology [8].

Recent studies examining the genomes and gene expression of phages and their bacterial hosts have shown that there is a close metabolic relationship between them [9,10]. Researchers have also discovered methyltransferases in some phages that are similar to the Dam methyltransferases found in the restriction modification systems of bacterial hosts. Although these methylases are more commonly found in temperate phages, it has been suggested that they may actually limit the lytic activity of the phages, rather than protecting their DNA from the endonucleases of the bacterial host [11]. This ongoing “arms race” between phages and bacteria has led to the development of adaptive cellular immunity systems like CRISPR/Cas in bacteria. Interestingly, analogues of these systems, which help phages protect themselves against bacterial defense systems, have also been discovered [12]. Tailed double-stranded DNA phages (dsDNA) from the *Caudoviricetes* class are the most common phages found in soil [13]. The best-described tailed phages infect Gamma-proteobacteria of the family Enterobacteriaceae, with over 31 identified species, including well-known model phages like λ, T4, T7, P2, P22, and Mu. Studies have shown that tailed dsDNA phage genomes are mosaic, meaning that there are identical blocks of genes in several phage genomes while numerous other blocks are individual. This confirms that tailed phages are actively involved in horizontal global gene transfer through specific and generalized transduction [14], which can lead to rearrangements of the bacterial genome.

Phages that integrate into the bacterial chromosome (prophages) can incorporate new genes that modify the virulence, toxin–antitoxin, or immune defense systems of bacterial hosts or change their metabolism [10,15,16,17]. In *E. coli* strains, it was shown that a single bacterial cell can contain up to 18 prophages simultaneously [18]. It is assumed that defective prophages that incorporate diverse foreign genetic sequences can also assist in diversifying the host bacterial genome [18,19]. Identifying novel phages can improve our understanding of phages’ role in horizontal gene transfer, bacterial ecology, and evolution.

Phages infecting root nodule bacteria (rhizobia), which are typical soil saprophytes capable of forming mutualistic nitrogen-fixing symbioses with legumes, have been less studied. Rhizobiophages of fast-growing and slow-growing species have been described to differ significantly in morphology, host bacterial spectrum, and general characteristics such as adsorption time, survival under different conditions, and restriction profiles [20,21]. Rhizobiophages mainly belong to the *Caudoviricetes* class according to classification [22], but they were previously assigned to the *Myoviridae*, *Siphoviridae*, and *Podoviridae* families as well [23,24,25].

Recently, phages of other species were identified, including *Inovirus* phages of the *Filamentous* family [21] and phages of the *Autographoviridae* family [26]. Rhizobiophages are abundant in various ecosystems. *Sinorhizobium meliloti* phages were isolated from soil where wild alfalfa grows, for example, phage P_ort11 was isolated from the rhizosphere of *Medicago marina* (Huelva, Spain) [21]. Some phages such as phiM7, PBC5, and HMSP1 were isolated from soil samples collected in agrocenoses, while other phages (phiM9, phiM10, phiM11, phiM12, and phiM14) were isolated from commercial alfalfa inoculants [21,27,28].

Unfortunately, full-genome sequences have been analyzed and published for only a limited number of rhizobiophages. There are data on 12 phages for *S. meliloti*, with genome sizes varying from 44 to 206.7 kb, differing in morphology and taxonomic affiliation (Table S1). Two well-studied phages are phiLM21 of *Sinorhizobium* spp. strain LM21, which was isolated from mineral sediments of Lubin copper mine (Poland) polluted by heavy metals [29], and the temperate *Rhizobium* phage 16-3, which was isolated using the *Rhizobium meliloti* 41 strain in 1961 in Hungary [30]. Physical and genetic maps for *Rhizobium* phage 16-3 were obtained in the 1970s–1980s, and the genome components have been studied in detail over the last 40 years: recombination system, major repressor protein C, *imm*X region responsible for the immune system, and tail complex proteins [2,3,4,30,31,32,33,34,35,36,37,38]. Recently, *Rhizobium* phage 16-3 was found in the virome of Lough Neagh lake and in groundwater from an urban area of Northern Ireland [39,40].

This article presents the genome of a new soil rhizobiophage, *Sinorhizobium* phage AP-16-3, which was isolated in the mountainous region of Dagestan, Caucasus, and found to be similar to *Rhizobium* phage 16-3. For the first time, the potential distribution of 16-3-like phages was evaluated using in silico analysis of prophage pools found in the genomes of *Sinorhizobium* spp. strains.

## 2. Materials and Methods

### 2.1. Bacterial and Phage Strains and Growth Conditions

The model prophage-free strain *Rhizobium meliloti* L5-30 (*Sinorhizobium meliloti* L5-30) was used to isolate bacteriophages according to [41]. The 18 randomly selected native strains were obtained from the laboratory collection of *S. meliloti* isolates of alfalfa from different far away geographical regions and used to test the lytic activity of bacteriophages. *S. meliloti* strains were cultivated on solid LB media (agar concentration 2%) at 28 °C. The same media with 0.6% agar was used as the top layer for titration of phages [42]. The spectrum of lytic activity of *Sinorhizobium* phage AP-16-3 was assessed according to the protocol described by [43,44,45].

### 2.2. Bacteriophage Isolation

Bacteriophage was isolated using an enrichment protocol [46] with some modifications. *Sinorhizobium* phage AP-16-3 was isolated from soil sample collected in mountainous region of the North Caucasus (Dagestan, Russia, 42°19′59.987″ N, 47°08′59.999″ E, Figure S1). For that purpose, 0.5 g of soil sample was added to 5 mL of LB and incubated for 2 h. Then, the soil suspension was filtered through a membrane filter with a pore size of 0.22 µm, and 300 µL of an overnight culture of *S. meliloti* L5-30 was added to the filtrate and incubated at 28 °C and 180 rpm for 2 h. Next, the mixture was poured into 1.5 mL microcentrifuge tubes, and 100 μL of chloroform was added, shaken for 5 min on a vortex, and centrifuged at 10,000× *g* for 10 min to remove bacterial fragments. Pure phage culture was isolated according to the temperature-adapted protocol of the agar double layer method developed by Adams [47].

In order to purify *Sinorhizobium* phage AP-16-3, the single plaque purification method was used [47]. A single plaque was removed from the top agar layer and added to 5 mL of LB medium and incubated for 2 h. Then, 300 µL of an overnight culture of *S. meliloti* L5-30 was added to the same tube, and the bacteriophage isolation protocol (described above) was followed. Passaging was carried out thrice for better purification of the phage [47].

### 2.3. DNA Isolation, Sequencing, and Annotation of the Bacteriophage Genome

Genomic DNA of phage AP-16-3 was isolated using the GeneJET Viral DNA/RNA Purification Kit. Genomic DNA was fragmented to an average size of 600 bp using the Covaris S2 instrument. A paired-end library was constructed using NEBNext dual-index oligonucleotide adapters and the NEBNext Ultra II DNA Library Preparation Kit for Illumina (New England BioLabs (NEB)). The DNA library was sequenced using the v3 reagent kit (2 × 300 bp) on a MiSeq desktop sequencer (Illumina) at the Genomics Center for Collective Use, Siberian Branch of the Russian Academy of Sciences, Novosibirsk (ICBFM Siberian Branch of the Russian Academy of Sciences) with a yield of about 1.4 million reads with paired ends. Short sequences were filtered for quality and adapter sequences were removed using the BBDuk software tool from the BBMap package (ktrim = r k = 23 mink = 11 hdist = 1 tpe tbo minlen = 25 qtrim = rl trimq = 10) with default parameters. Annotation of the bacteriophage genome was performed using eggNOG-mapper v2 [48].

### 2.4. The Search of Phage-like Sequences

The search for phage-like sequences was performed in the genomes of 38 strains of *Sinorhizobium* spp. (Table S2) and in the genomes of strains of *Rhizobium lentis* BLR27, *Rhizobium* sp. NLR16a, *Rh. leguminosarum* OyaliB, and *Mesorhizobium* sp. DCY119 (Access date September 2022; Table S2) using the PHASTER web server [49], the Prophage Hunter [50], and IslandPath-DIMOB (for *S. meliloti* prophage NV1.1.1) [51], and was manually curated.

### 2.5. Analysis of Homology, Codon Usage, tRNA, and Pairwise Comparison Analysis of Nucleotide and Amino Acid Sequences

The search for tRNA genes in prophage sequences was performed using tRNAscan-SE [52]. Programs from the BLAST family were used to identify and analyze nucleotide and amino acid sequences, respectively, based on their similarity to database sequences. Codon counts were determined using Sequence Manipulation Suite: Codon Usage [53]. Codon usage frequencies were determined as the proportion of the use of a specific codon of a certain amino acid from all codons of this amino acid. Pairwise comparison analysis of phages and prophages was performed using VIRIDIC [54]. The comparative alignment of phage sequences was visualized using Easyfig [55] and Proksee server (https://proksee.ca/ (accessed on September 2022)). Alignment of phage genomes was performed using Mauve 2.4.0 (The Darling Lab, Sydney, Australia).

### 2.6. Phylogenetic Analysis

Multiple nucleotide sequence alignment was performed using Muscle tool [56]; highly variable fragments of the alignments were eliminated using Gblocks program [57]; phylogenetic analysis was conducted using IQ-TREE [58] with the maximum likelihood algorithm (1000 bootstrap replicates), and the trees were rendered with Dendroscope3 [59]. The relative rate of accumulation of substitutions (N) between sequences were calculated by using the formula: N = L/100 × n, where L is the sequence length, and n is the number of substitutions per 100 nucleotides. Protein structure homology modelling, such as identification of structural template(s), alignment of target sequence and template structure(s), model-building, and model quality evaluation were performed using web-based integrated service SWISS-MODEL [60]. The accuracy of the tertiary structure of the resulting model was estimated by average GMQE (Global Model Quality Estimate) with standard deviation.

### 2.7. Nucleotide Accessions

The genome sequences of AP-16-3 and NV1.1.1 were deposited in the GenBank database with the nucleotide accession numbers OP484857 and OP484858.

## 3. Results

### 3.1. Biological and Molecular Genetic Characteristics of Phage AP-16-3

**Figure 1:**
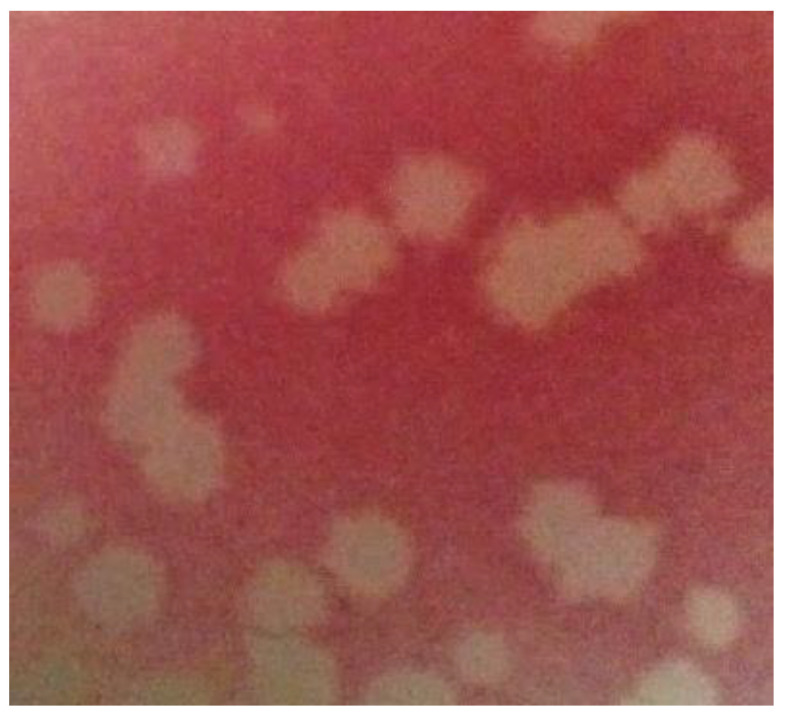
Lysis plaques of *Sinorhizobium* phage AP-16-3 observed in *S. meliloti* L5-30 using the double-layer agar method.

Phage AP-16-3 was isolated from a soil sample collected in the mountainous area in the North Caucasus, which is part of the primary legume diversity center (see Section 2, Figure S1). The *S. meliloti* L5-30 strain was used to isolate the AP-16-3 phage, but this strain did not appear to be the ideal host for maintaining the viability of our isolated phage, as its titer declined sharply after 4 months of storage at −80 °C. This low viability of the phage is consistent with the data [61]. This phage, named *Sinorhizobium* phage AP-16-3, formed transparent negative colonies 3–5 mm in diameter on a turf of bacterial cells (Adams method) (Figure 1). This phage demonstrated selective lytic activity, lysing only 3 of the 18 tested native *S. meliloti* strains obtained from geographically distant regions.

**Figure 2:**
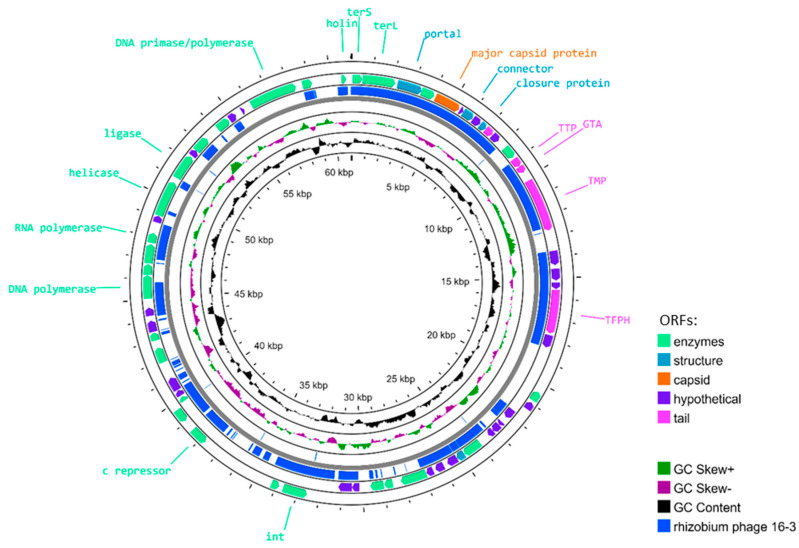
Physical map of the *Sinorhizobium* phage AP-16-3 genome. Circles show (from the outside to the inside scaled in kb): (1) ORFs transcribed in the clockwise or counterclockwise direction. ORFs encoding different proteins are in different colors. (2) Regions homologous to *Rhizobium* phage 16-3. (3) GC skew (G − C/G + C, in a 1 kb window with a 0.1 kb incremental shift). Values greater than zero are in green, while those lower than zero are in purple. (4) G + C% content (in a 1 kb window with a 0.1 kb incremental shift). Values greater than 49.42% (average) are towards the outside, while values lower than 49.42% are towards the inside.

**Figure 3:**
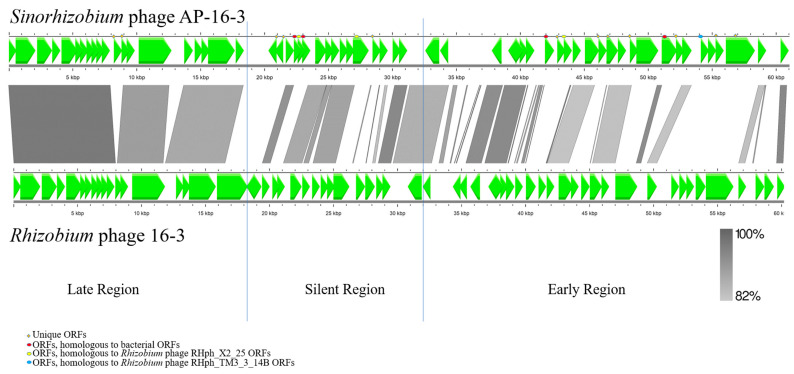
Alignment of genome sequences of *Sinorhizobium* phage AP-16-3 and *Rhizobium* phage 16-3. Areas with homology from 82% to 100% are highlighted in gray. Late, silent, and early regions of viral genes are defined according to [3,62]. The late region (L) of AP-16-3 starts from ORFs encoding the large and small terminase subunits and ends with ORFs encoding a chitinase-like protein (*Eco*RI restriction site, 16,823 bp; annotation according to the PHASTER algorithm). The conditional boundary between the silent region (S) and the early region (E) is located between the ORFs encoding the excisionase and the repressor protein.

The genome of phage AP-16-3 (dsDNA) is 61,037 bp, 800 bp greater than that of *Rhizobium* phage 16-3 (Table 1). The constructed physical map of the AP-16-3 genome is presented in Figure 2. The genome of AP-16-3 had 55% nucleotide sequence coverage and 96.18% identity with the genome of *Rhizobium* phage 16-3 (NCBI RefSeq: NC_011103.1) according to BLASTn analysis. The GC content in AP-16-3 was 59.22%, which was slightly higher than that of 16-3 (Table 1). Alignment of the nucleotide sequences of both phages revealed 32 homologous regions with a total length of 45,782 bp, indicating the mosaic structure of the AP-16-3 phage genome with respect to *Rhizobium* phage 16-3 (Figure 3).

AP-16-3 DNA was analyzed considering the restriction sites of various endonucleases, as in a previous study on tailed phages [20]. The genomes of both phages contained an excessive number of sites for the endonucleases *Hae*III, *Taq*I, and *Hha*I (about 400 sites in the case of the first two endonucleases, and more than 700 sites in the latter case, Table S3) and did not contain sites for *Bfo*I and *Rsa*II. The AP-16-3 phage genome contained 1.6-fold more sites for *Eco*RI and 3–10% more sites for *Alu*I, *Hha*I, and *Taq*I, whereas the number of sites for *Bsu*RI and *Hpa*II was 3–6% lower than the number of sites for the above endonucleases in phage 16-3 (Table S3).

The genome of phage AP-16-3 contained 62 ORFs (open reading frames, orphan genes), as in phage 16-3, but only 42 ORFs were homologous to each other (according to eggNOG-mapper; Table 1). ORFs encoding capsid, tail, portal, and adapter proteins were identified in the genomes of both phages, as well as ORFs of hypothetical phage proteins and ORFs encoding bacteriophage-specific enzymes, such as terminase (small and large subunits), helicase, integrase, excisionase, and choline, responsible for interaction with the host cell and insertion into and release from the genome (Table S4). The listed terminases and choline were also detected in strains of *Rhizobium* spp. The presence of integrase and excisionase in the AP-16-3 genome suggests that this is a temperate phage. AP-16-3 contains an *imm*C region element (ORF 39), which confers immunity to homoimmune infections to the transductant Rm41, according to [3] (Table S4). Amino acid sequence analysis of 15 ORF products showed that they were homologous to both phages analyzed (according to eggNOG-mapper v2), but homology with other phages, like *Rhizobium* phages RHph_X2_25 (NCBI RefSeq: MW960032.1) and RHph_TM3_3_14B (NCBI RefSeq: MN988496.1) from the class *Caudoviricetes* and isolated in Mexico was observed. The phage AP-16-3 contained seven ORFs (32, 33, 44, 49, 53, 57 и 60; Table S4) encoded *Sinorhizobium/Rhizobium* species proteins classified as EC 2, EC 3, EC 5.6, EC 7 of NC-IUMBMB (https://iubmb.qmul.ac.uk/enzyme/ (accessed on 26 July 2023)). Besides that there were five ORFs encoded bacterial proteins that were homologous to hypothetical proteobacterial proteins (ORFs 22 and 24), the FkbM family methyltransferase from *S. fredii* (ORF 21), an alphaproteobacteria-specific ligase (ORF 54), the DCL family protein of *S. meliloti* (ORF 59), which was also similar to proteins from other bacterial species (*Ktedonobacter robiniae*, identity = 51.85%, cover = 98%), and eukaryotes (identity = 50–52.73%, cover = 98–100%; Table S4).

Rhizobiophage AP-16-3 is related to *Rhizobium* phage 16-3 isolated from soil with *S. meliloti* Rm41 in Hungary in 1961 [30]. Rm41 whole-genome sequence data were deposited in the NCBI database twice, once in 2012 (BioSample SAMEA2272434) and again in 2017 (SAMN07175165) (Table S2). Analysis of both annotations showed that there were four phage sequences in chromosome Rm41. Two sequences were intact prophages related to phiLM21, but both contained ORFs 1 and 2 homologous to phage 16-3 (Table S2). The other two sequences were incomplete prophages related to *Enterobacteria* phages. No extended sequences related to *Rhizobium* phage 16-3 or sequences of the *imm*C region were found in both deposited Rm41 genome sequences (hereafter considered BioSample SAMN07175165; Table S2).

### 3.2. Prophages in Rhizobia Genomes

**Figure 4:**
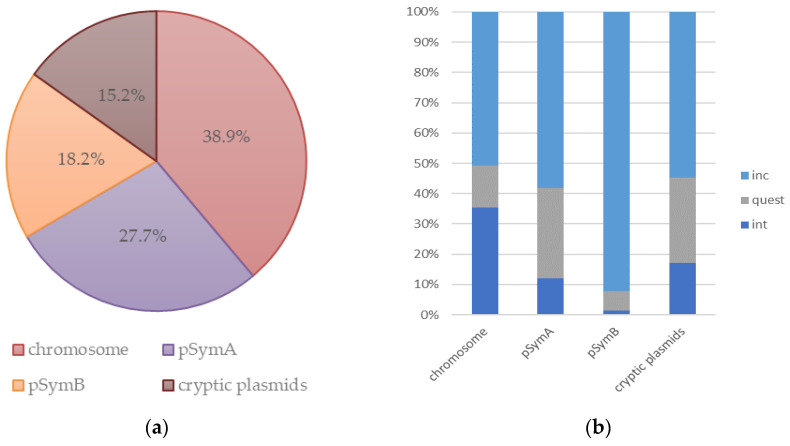
PhRSs in the genomes of *Sinorhizobium* spp.: (**a**) frequency of occurrence of PhRSs on the chromosome and plasmids; (**b**) frequency of intact, incomplete, and questionable PhRSs on the chromosome and plasmids.

A search for sequences related to phages AP-16-3 and 16-3, whose genomes are mosaic, was performed in the genomic sequences of 38 strains of *Sinorhizobium* spp. (Table S2). A total of 422 prophages were identified using the PHASTER web server in the genomes of 31 strains of *S. meliloti*, and in the genomes of two and five strains of *S. medicae* and *S. fredii*, respectively. It was shown that 4 to 30 phage-related sequences (hereafter, PhRSs) can be present in the genome of *Sinorhizobium* spp. simultaneously, with an average of 11 PhRSs per genome (Table S2, Figure 4a). In the studied strains, prophages were predominantly localized on the chromosome (frequency of 0.39) but were also found on both megaplasmids (Figure 4).

At the same time, PhRSs were present one and a half times more frequently on pSymA carrying *nod*/*nif*/*fix* genes than on pSymB (0.28 and 0.18, respectively) or cryptic plasmids (0.15). The size of the PhRSs ranged from 3.2 to 70.4 kb, and their GC% content ranged from 51.42 to 65.06%. PhRSs showed homology with 64 phage species and 36 unknown phages belonging to the *Caudoviricetes* class (according to PHASTER and ICTV 2022 taxonomy). It should be noted that up to 10 PhRSs belonging to different phage species can be present in the genome of a single strain simultaneously. PhRSs identified in *Sinorhizobium* spp. strains were represented by intact phages (frequency of 0.20) and incomplete and questionable sequences (0.61 and 0.19, respectively).

The composition of all three types of phage-related sequences was most common in the genomes of *Sinorhizobium* spp. (frequency of 0.61). Intact prophages were mostly integrated in the chromosome of the studied strains (frequency of 0.35) and often homologous to the *Sinorhizobium* phiLM21 phage of the *Caudoviricetes* class (frequency of 0.32) described in 2014 [29]. Incomplete prophages were predominantly found on the pSymB megaplasmid (frequency of 0.92) (Figure 4b).

#### Prophages Containing ORFs Homologous to AP-16-3 and 16-3 Phages

Open reading frames (ORFs) homologous to *Rhizobium* phage 16-3 were identified in 39 prophages of the 422 PhRSs of *Sinorhizobium* spp. mentioned above (hereafter PhRS-16-3; Table S2). These 39 sequences were divided into two groups of prophages. The first group included phage sequences identified as *Rhizobium* phage 16-3-like using PHASTER (hereafter PhRS-like-16-3). The second group included phage sequences that contained no more than four ORFs homologous to *Rhizobium* phage 16-3 (hereafter, PhRS-orph-16-3). The detected PhRS-16-3 was predominantly localized on the chromosomes of the studied strains, except in a few cases where PhRS-orph-16-3 was localized on other replicons, namely, on pSymB (*S. meliloti* strains USDA1021 and USDA1157) and on the non-symbiotic plasmid pSF8366d (size 76,753 bp) of *S. fredii* strain CCBAU 83666 (Table S2). It should be noted that prophages from both of these groups, PhRS-like-16-3 and PhRS-orph-16-3, as well as other unrelated phage sequences, can be present simultaneously in the same genome, as in the case of *S. medicae* strain WSM419, which has 13 different PhRS sequences present in its genome simultaneously (Table S2).

Thirty-three prophages were assigned to the PhRS-orph-16-3 group, of which more than half (frequency of 0.58) were related to *Sinorhizobium* phage phiLM21, and the remaining sequences were related to other phages, including *Sinorhizobium* phage phiM7, *Rhizobium* phage vB_RleM_PPF1, *Mesorhizobium* phage vB_MloP_Lo5R7ANS, *Brucella* phage BiPBO1, *Enterobacteria* phage mEp235, *Pelagibacter* phage HTVC010P, *Sulfitobacter* phage NYA-2014a, and *Rhizobium* phage 16-3 mentioned above. Analysis of the nucleotide sequences of 18 intact PhRS-orph-16-3s, which were related to phiLM21, showed that their homology level with this phage did not exceed 37.9%, whereas between the prophages themselves, the similarity level reached 95.6% according to a pairwise comparison of the nucleotide sequences (Figure S2).

**Figure 5:**
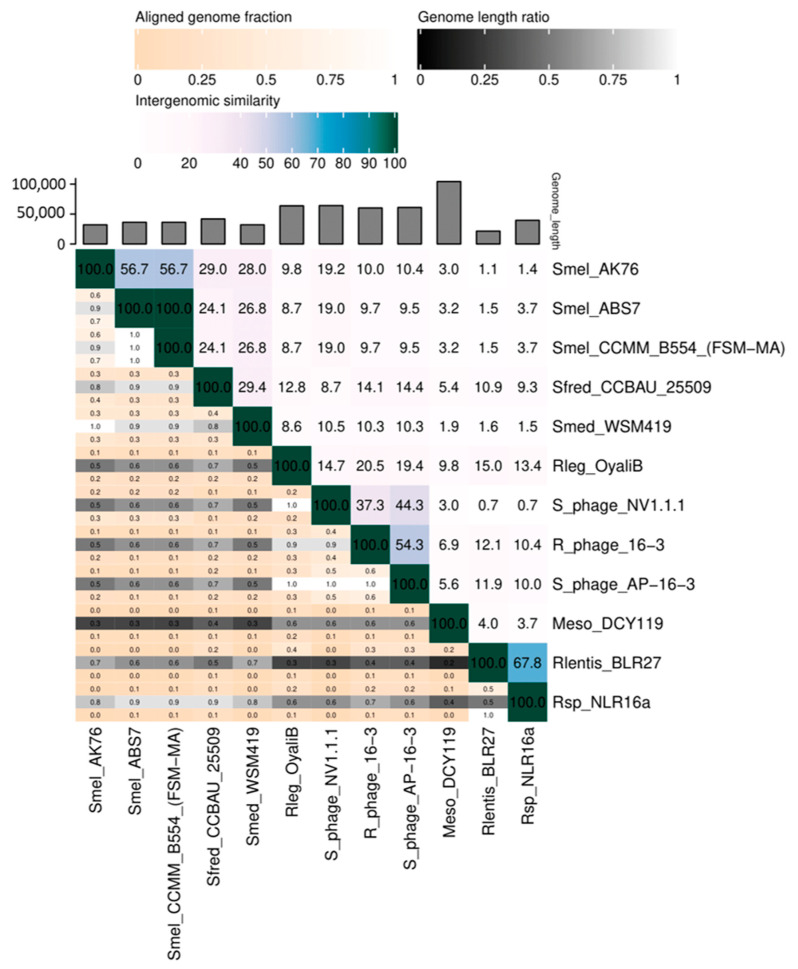
Intergenomic similarity values (right half) and alignment indicators (left half and top annotation) between *Sinorhizobium* phage AP-16-3, *Rhizobium* phage 16-3, and prophages (PhRS-16-3-like) identified in rhizobia strains according to VIRIDIC [54]. In the right half, the numbers represent the similarity values for each genome pair. In the left half, three indicator values are presented for each genome pair, in the order from top to bottom: aligned fraction genome 1 (for the genome found in this row), genome length ratio (for the two genomes in this pair), and aligned fraction genome 2 (for the genome found in this column).

**Figure 6:**
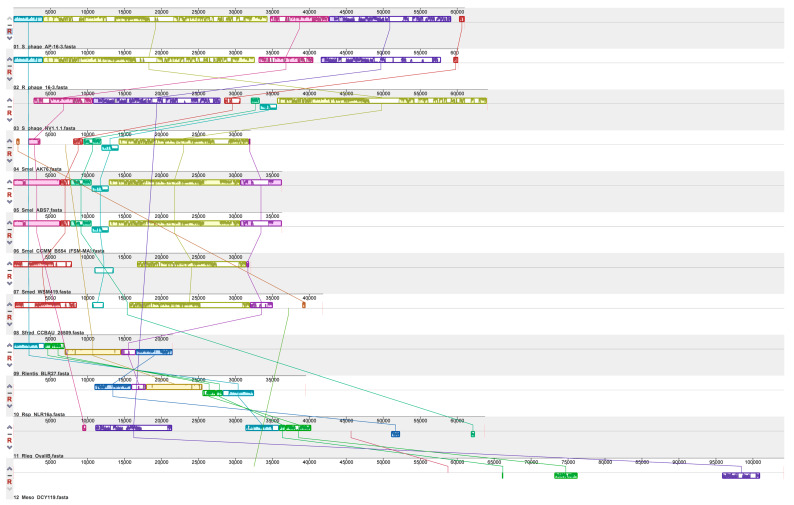
Sequence alignment of *Sinorhizobium* phage AP-16-3, *Rhizobium* phage 16-3, and prophages (similar to PhRS-16-3) identified in rhizobia strains. Colored lines indicate which regions in each sequence are homologous.

The PhRS-like-16-3 group included six prophages that were identified in the genomes of four *S. meliloti* strains as well as in the *S. medicae* WSM419 and *S. fredii* CCBAU 25509 strains (Table 2 and Table S1). Twelve to sixteen ORFs homologous to phages AP-16-3 and 16-3 were present in five PhRS-like-16-3s according to PHASTER analysis (Table 2 and Table S2). The sequences of intact prophages in the *S. meliloti* strains CCMM B554 (FSM-MA) and ABS7 were homologous to each other, were of equal size (36.3 kb) (similarity = 100%; Figure 5), and had a high level of similarity to the prophage of the *S. meliloti* strain AK76 (similarity = 56.7). Meanwhile, the intact prophage of *S. medicae* WSM419 (32.1 kb) and the prophage of *S. fredii* CCBAU 25509 (41.8 kb) had a similar but significantly lower level of homology both among themselves (similarity = 29.4%) and with the above-mentioned *S. meliloti* prophages (mean similarity = 26.6%) (Figure 5). In the *S. fredii* and *S. medicae* prophages, an ORF homologous to that in AP-16-3, which was also present in phage RHph_X2_25 but was absent from the *Rhizobium* phage 16-3 genome, was detected. The tRNA-Met gene was identified in *S. fredii* prophage CCBAU 25509. All identified intact prophages in the *Sinorhizobium* spp. strains had extended homologous regions with phages AP-16-3 and 16-3, but these regions were inverted compared to the sequences of the phages compared (Figure 6). Only the prophage of the *S. meliloti* strain NV1.1.1 revealed 51 ORFs homologous to phages AP-16-3 and 16-3 according to PHASTER analysis. The strain NV1.1.1 was isolated from a soil sample from the Shalkar District (Mugodzhar mountain region) in northern Kazakhstan belonging to the modern center of introgressive hybridization (unpublished data of V. S. Muntyan; Table S2, Figure S1).

The intact prophage NV1.1.1 was 64.2 kb in size, with a GC% content of 59.4%, and its sequence was 44.4% and 37.4% similar to AP-16-3 and phage 16-3, respectively (Figure 5). Prophage NV1.1.1 had *att* sites, a tRNA-fMet gene, and 51 ORFs encoding proteins homologous to phage 16-3 proteins, as annotated by PHASTER (Table 2). The sequences of prophage NV1.1.1 and phage AP-16-3 had a total of 33 mosaically arranged sequences, which were 82% to 100% identical (BLASTn). Analysis of the amino acid codons encoded by *S. meliloti* NV1.1.1 prophage genes, as well as by phage AP-16-3 and *Rhizobium* phage 16-3, showed that these sequences did not differ in the predominance of the different amino acid codons used (*p* > 0.05), which allowed us to consider them as sequences of related origin.

Interestingly, PhRS-like-16-3 was detected in the genomes of nodule bacteria from two other genera of the order *Hyphomicrobiales* using BLAST family programs. These prophages were detected on the chromosomes of strains *Rh. leguminosarum* OyaliB, *Rhizobium lentis* BLR27, *Rhizobium* sp. NLR16a, and *Mesorhizobium* sp. DCY119 (PHASTER analysis), of which three were intact and had *att* sites (Table 2; Table S2). The last three listed PhRS-like-16-3 contained 12 to 14 ORFs of interest. More than half of the *Rhizobium* sp. NLR16a prophage sequence was homologous to the *Rh. lentis* strain BLR27 (Cov = 99%, E-value = 0.0, identity = 96.70%, according to BLASTn), and the intergenomic similarity between them was 67.8% (Figure 5). The intergenomic similarity between *Rh. lentis* BLR27, *Rhizobium* sp. NLR16a, and *Mesorhizobium* sp. DCY119 with phage sequences AP-16-3 and 16-3 did not exceed 12.1%, and their similarity to *Sinorhizobium* spp. was lower (10.9%) (Figure 5).

Prophage *Rh. leguminosarum* OyaliB had 39 ORFs homologous to phages AP-16-3 and 16-3 (according to PHASTER), and tRNA-Lys and *tfp*H genes have been also found in prophages of *Sinorhizobium* spp. The similarity level of the *Rh. leguminosarum* OyaliB prophage was higher with phage 16-3 than with phage AP-16-3 (20.5 and 19.4%, respectively), whereas the similarity level of the *S. meliloti* NV1.1.1 prophage was, conversely, higher with phage AP-16-3 than with phage 16-3 (44.3% and 37.3%, respectively) (Figure 5). The prophage from *Rh. leguminosarum* OyaliB showed the highest level of similarity with the prophages from *S. fredii* CCBAU 25509 and *Rh. lentis* BLR27 (12.8 and 15%, respectively), whereas the *S. meliloti* prophage NV1.1.1 had a higher similarity with the above-discussed prophages from *S. meliloti* strains (19% on average). The level of similarity between the *Rh. leguminosarum* OyaliB and *S. meliloti* NV1.1.1 prophages did not exceed 14.7% (Figure 5).

**Figure 7:**
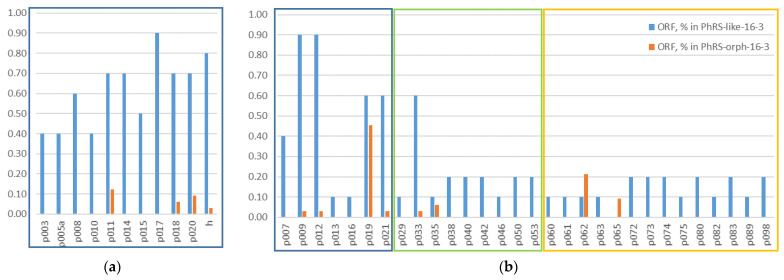
Frequency of occurrence of ORFs in PhRS-like-16-3 and PhRS-orph-16-3 prophages and their correlation with the L, S, and E regions of AP-16-3 phage (marked in blue, green, and orange, respectively): (**a**) ORFs encoding the structural elements of a viral particle; (**b**) ORFs encoding hypothetical proteins.

An analysis of 39 prophages from both PhRS-like-16-3 and PhRS-orph-16-3 identified 249 phage ORFs that were homologous to 57% of the AP-16-3 phage ORFs (PHASTER annotation). All of the indicated ORFs belonged to the regions of late (L), silent (S), and early (E) viral genes (Figure 7). In the PhRS-like-16-3 prophage group, there were 11 ORFs encoding virion elements, 29 ORFs encoding hypothetical proteins, and 21 ORFs encoding enzymes, all of which were homologous to phage AP-16-3.

In the late viral gene region (L) of phage AP-16-3, there was an alternation of ORFs encoding virion elements. This included the following ORFs: head closure protein (p010), tail tube protein (p014), tail length tape measure (p017), tail fiber protein H (h), undifferentiated tail proteins (p011, p018, p020), p015, encoding gene transfer agent family protein, and ORFs that determine the synthesis of hypothetical proteins (p012, p013, p016, p019, and p021) (Figure 7a). A similar sequence of ORFs was detected in the PhRS-like-16-3 prophages of *Sinorhizobium* spp. strains as well as in the prophage from *Rh. leguminosarum* OyaliB. This group of ORFs consisted of the above seven ORFs encoding different tail proteins, which alternated with ORFs encoding hypothetical proteins (Figure 7). At the same time, some of these ORFs were not present in the investigated prophages. For example, p015, p010, and orphans p013 and p016 were present only in prophage NV1.1.1 (Table 3). The orphan gene p005a, which determines capsid synthesis, together with two adjacent orphan genes, was present only in the prophages from *S. fredii*, *Rh. lentis*, *Rh*. spp., and *Rh. leguminosarum* OyaliB (Table 3). In an analysis of 33 PhRS-orph-16-3 prophages, seven ORFs were identified that belonged to the LR region of phage AP-16-3 (Figure 7). These single orphan genes were present in only 8 out of 33 prophages, of which 4 prophages were intact. The exception was the prophage from USDA1157 localized on pSymB, which contained four orphan genes, three of which encoded virion structural elements (p018, p020, p022 (h)), with the remaining orphan gene p021 encoding a hypothetical protein that belonged to the L region of phage AP-16-3. Of particular note is the orphan gene p019, which encoded a hypothetical protein that, according to our analysis, presumably encodes the METTL16 domain, which is the long-unknown methyltransferase for the U6 spliceosomal small nuclear RNA (snRNA) [63] and has been shown to display nuclear localization [64]. The orphan gene p019 was detected in a single copy in 13 intact PhRS-orph-16-3 prophages (frequency of 0.56), including 12 phiLM21-like prophages, and in the *Pelagibacter* phage HTVC010P-like prophage identified in *Sinorhizobium* spp. strains. This orphan gene was represented by two copies in each of the three prophages of the two studied *S. medicae* strains.

ORFs that encode hypothetical proteins are the most numerous and diverse group. The analysis of PhRS-like-16-3 and PhRS-orph-16-3 prophages revealed 30 ORFs and 8 ORFs, respectively. The greatest numbers of orphan genes were detected in the prophages of *Sinorhizobium* spp. NV1.1.1 and *Rh. leguminosarum* OyaliB, with 26 and 13, respectively, whereas an average of 4.37 orphan genes were present in the remaining PhRS-like-16-3 prophages (a total of 74 ORFs). In total, four of the eight ORFs of the PhRS-orph-16-3 prophages belonged to the LR region of phage AP-16-3. The remaining four ORFs were found in single copies in nine different PhRS-orph-16-3 prophages. Only the orphan gene p062 was present in six intact, incomplete, and questionable sequences and detected in two copies in the prophage of strain HM006 (Figure 7b).

A third group of ORFs encoded enzymes involved in information storage and processing and metabolism, as well as those responsible for the process of interaction with the host cell, for the release of the viral particle from the cell, and for endo/exonuclease synthesis. Twenty-one ORFs were identified in the PhRS-like-16-3 prophages. The greatest equal number of the indicated ORFs (17 ORFs each) was present only in the prophages of *Sinorhizobium* spp. NV1.1.1 and *Rh. leguminosarum* OyaliB, while the other prophages contained no more than four ORFs. The analysis of the PhRS-orph-16-3 prophages revealed only two ORFs homologous to phage AP-16-3, which were responsible for interaction with the host bacterium and belonged to the early (E) viral gene region of the phage. These were orphan p097, presumably encoding DNA methylase, which was present in the prophages of *S. meliloti* and *S. medicae* strains, and orphan p067, the product of which was the C-repressor protein, identified in ten intact and incomplete prophages.

**Figure 8:**
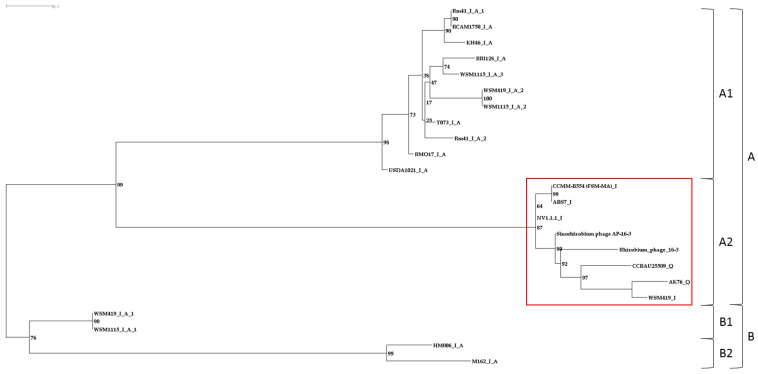
Phylogenetic tree constructed on ORF p019 of *Sinorhizobium* phage AP-16-3 and *Rhizobium* phage 16-3 and prophage sequences (PhRS-16-3) of *Sinorhizobium* spp. The nucleotide substitution model selected for the analysis was TIM3e + G4. The scale bar is 0.01 for nucleotide substitutions per site (see Section 2). Cluster PhRS-like-16-3 sequences marked in red. A and B–clades; A1, A2, B1 and B2–clusters.

Consequently, the PhRS-like-16-3 and PhRS-orph-16-3 prophages contained ORFs encoding hypothetical proteins, which were the most abundant in both of these groups of prophages. ORFs encoding virion elements of phage AP-16-3 and enzymes important for virion–host cell interaction were found in PhRS-like-16-3, with 6.8 and 5.5 ORFs per prophage on average, whereas in PhRS-orph-16-3, similar ORFs were found more than 20 times less frequently. It should be noted that an ORF encoding the C-repressor protein and orphan p011 encoding a tail protein (E and L regions, respectively) were found in one-third of the analyzed PhRS-16-3 prophages, whereas an ORF encoding the structural protein *tfp*H of the virus particle used in the phylogenetic studies was found significantly less frequently (0.21). In both prophage groups, namely, PhRS-like-16-3 and PhRS-orph-16-3, the most frequent was ORF p019, which presumably encoded a hypothetical protein that is a domain of the METTL16 protein (frequencies of 0.6 and 0.48, respectively). The consensus sequence size of the p019 gene was 118 bp. Phylogenetic analysis of the 23 p019 sequences revealed two clades, A and B, with two clusters each (bootstrap 99% and 76%, respectively; Figure 8). Cluster A1 included 11 p019 gene sequences present in phiLM21-like phages, whereas cluster A2 included similar sequences present in six PhRS-like-16-3 phages as well as in AP-16-3 and 16-3 phages. Clade B contained p019 sequences identified in the *Pelagibacter* phage HTVC010P-like prophage and in three phiLM21-like prophages of *S. meliloti* and *S. medicae* strains. The relative rate of accumulation of substitutions between putative ancestral sequences for these clusters was 0.8208783752 bp for every 100 bp of sequence. Comparative analysis of the topology of the trees constructed from nucleotide and amino acid sequences revealed no significant difference in the grouping of sequences into clades; however, the topology was different, suggesting functional divergence of the amino acid sequences (V.S. Muntyan, unpublished data). The products of the p019 gene sequences grouped in cluster A1 had a monomeric secondary structure, whereas those of the p019 gene sequences grouped in cluster A2 had monomeric or heterodimeric proteins in equal proportions (50:50%) and belonged to different domains of methyltransferase-like protein 16 (METTL16) (GMQE (Global Model Quality Estimate) = 40.41 ± 2.3%, calculated using SWISS-MODEL) [60]. Thus, the sequences encoding the putative METTL16 protein domains identified in PhRS-like-16-3 phages were phylogenetically related to the sequences of AP-16-3 and 16-3 phages encoding a similar protein. Meanwhile, the sequences identified in other PhRS-16-3 prophages had distant phylogenetic origins and possibly encode protein domains of a different functional significance.

**Figure 9:**
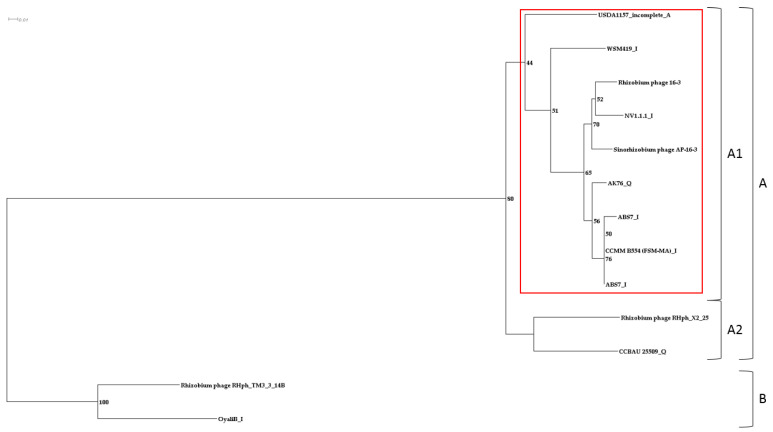
Phylogenetic tree constructed on *tfp*H sequences of *Sinorhizobium* phage AP-16-3 and *Rhizobium* phage 16-3 and prophage sequences (PhRS-16-3) of *Sinorhizobium* spp. The nucleotide substitution model selected for the analysis was TN + F + G4. The scale bar is 0.1 for nucleotide substitutions per site (see Section 2). I—intact prophage; Q—questionable prophage; A—prophage annotated using the PHASTER algorithm as different from *Rhizobium* phage 16-3; xx—region number, according to PHASTER data; yy—serial number of the copy in the prophage sequence. Cluster PhRS-like-16-3 sequences marked in red. A and B–clades; A1, A2, B1 and B2–clusters.

Phylogenetic analysis of PhRS-like-16-3 prophages was performed using the sequence of the *tfp*H gene encoding putative tail fiber protein H. This protein belongs to the group of proteins of the distal part of the tail fibers of *Caudoviricetes* class viruses. The fibers are responsible for specific, although reversible, primary attachment to the host bacterial cell [65,66]. The *tfp*H gene sequence was detected in six PhRS-like-16-3 prophages from *Sinorhizobium* spp. strains (Table 2) and in the *Rh. leguminosarun* OlyahB-1 prophage. Similar sequences detected in the phiLM21-like prophage of the *S. meliloti* USDA1157 strain, as well as in the tail rhizobiophages RHph_X2_25 and RHph_TM3_3_14B from the *Caudoviricetes* class isolated from soils in Mexico [67,68], were also included in the analysis. The *tfp*H gene sequences of phages AP-16-3 and 16-3 were 2106 and 2112 bp, respectively, and had a similarity of 89.59% (100% coverage). In the above strains, the size of the *tfp*H gene ranged from 2004 bp to 2124 bp, and the length of the aligned consensus sequence was 2105 bp (see Section 2). Cluster A1 combined five sequences of the *tfp*H gene from PhRS-like-16-3 phage strains of *S. meliloti* and *S. medicae*, as well as similar sequences of phages AP-16-3 and 16-3. The *tfp*H gene sequences of *Rhizobium* phage RHph_X2_25 and the prophage of *S. fredii* strain CCBAU 25509 were grouped into cluster A2 (bootstrap 80%, Figure 9). Cluster B combined the *tfp*H gene sequences of *Rhizobium* phage RHph_TM3_3_14B and prophage *Rh. leguminosarum* OyaliB (Cov = 98%, E-value = 2 × 10^−69^, identity = 63.64%). The *tfp*H gene sequence of phage AP-16-3 is phylogenetically closer to the analogous sequence of *Rhizobium* phage RHph_X2_25 than to that of *Rhizobium* phage RHph_TM3_3_14B. This is confirmed by the sequence identity assessment of the corresponding *tfp*H genes of the RHph_X2_25 and RHph_TM3_3_14B phages (identity = 80.83%, Cov = 99%, E-value = 0.0, and identity = 63.64%, Cov = 98%, E-value = 2 × 10^−69^, respectively).

According to the evaluation of the relative accumulation rate of nucleotide substitutions in the studied bacteriophages, it was concluded that *Sinorhizobium* phage AP-16-3, *Rhizobium* phage 16-3, and *Rhizobium* phage RHph_X2_25 had the most recent common ancestor (MRCA) of the nucleotide sequence of the *tfp*H gene (2.7916234245 bp and 7.8451268576 bp for every 100 bp relative to AP-16-3, respectively), while the corresponding phage RHph_TM3_3_14B sequence was further away (35.9173312881 bp for every 100 bp).

## 4. Discussion

This work presents, for the first time, a systematic analysis of phage sequences in the genomes of strains of *Sinorhizobium* spp. root nodule bacteria and representatives of other rhizobia genera, which are non-obligate nitrogen-fixing symbionts of forage legume grasses. The genome of *Sinorhizobium* phage AP-16-3 from soils of the mountainous area of the center of diversity of cultivated plants in the Caucasus was homologous to that of phage 16-3. The structure of phage AP-16-3 was characterized as mosaic relative to *Rhizobium* phage 16-3. The nucleotide sequences of the phages showed a similarity of 55%. Pairwise comparison of amino acid sequences (BLASTp) of phage ORFs showed that 72.6% of the sequences are homologous between phages. For the first time, the existence of a putative ancestral phage sequence was predicted for *Sinorhizobium* phage AP-16-3 and *Rhizobium* phage 16-3 as well as for *Rhizobium* phage RHph_X2_25, all of which had different geographically distant origins (Dagestan, Hungary, Mexico). This study allowed us, for the first time, to estimate the distribution potential and viability of temperate tailed rhizobiophages. It was shown that strains of *Sinorhizobium* spp. can contain up to 30 prophages per genome, with the insertion of phage sequences occurring predominantly in the chromosome (frequency of 0.39). It was shown that the phage sequences were also actively inserted into a symbiotic megaplasmid (pSymA), but 1.4 times less frequently.

Interestingly, phage sequences were identified on the pSymB megaplasmid and on cryptic plasmids with a similar frequency (0.16, on average). The fact that intact prophage sequences were quite often found in cryptic plasmids (frequency of 0.17) makes the study of cryptic plasmids as selective objects of phage integration for the purposes of agrobiotechnology a promising field. The combination of phage sequences of different completeness in the same genome was typical for *Sinorhizobium* spp. strains. The sequences detected were related to 100 different phages, while the rest belonged to 23 genera, 9 subfamilies, and 11 different families according to the NCBI data. These phage sequences have been identified in various representatives of alpha-, beta-, and gamma-proteobacteria (*Gammaproteobacteria*), bacilli (*Bacillota*), actinomycetes (*Actinomycetota*), cyanobacteria (*Cyanobacteriota*/*Melainabacteria* group), and archaea. The analysis of *Sinorhizobium* spp. genomes showed that they could simultaneously contain phage sequences related to at least 10 phylogenetically different phages. The data presented are in agreement with those published by other authors [69] and clearly demonstrate the scale of the infectious activity of phages and the potential possibility of horizontal transfer of foreign genes.

According to our analysis of prophages in rhizobia strains presented in the NCBI database, the number of characterized bacteriophages for *S. meliloti* is very limited. In an analysis of 422 prophages of *Sinorhizobium* spp., the temperate phages phiM7, phiM9, phiN3, and phiLM21 and phage 16-3 discussed here were identified. We report here that sequences related to *Sinorhizobium* phage phiLM21 were found in the genomes of more than half of the *Sinorhizobium* spp. strains studied (63%), which is consistent with the data we obtained earlier when *S. meliloti* strains were studied [70]. Phage phiLM21 of the *Caudoviricetes* class was described as a unique phage not related to known phages [29]. The data presented here provide indirect evidence of the high infectivity of phage phiLM21 against geographically diverse strains of *Sinorhizobum* spp., which, in turn, allows us to conclude that a significant proportion of *Sinorhizobium* spp. strains apparently did not previously have an immune defense system against this newly described phage. The PhRS-like-16-3 and PhRS-orph-16-3 sequences studied were represented predominantly by intact prophages (72%), which allowed us to conclude that phages related to phage 16-3 had broad infectivity against rhizobia of different species and genera. Rhizobia strains in which prophages related to AP-16-3 were detected belonged to different species, genera, and even families and had different geographic origins. Thus, the *S. meliloti* NV1.1.1 strain was isolated from saline soils in northern Kazakhstan, the *Rh leguminosarum* OyaliB strain was a symbiont of *Lens culinaris* growing in Turkey (BioProject PRJNA623125, [71]), and *Mesorhizobium* sp. DCY119 (*Mesorhizobium panacihumi* genotype), which contained a prophage with a significant number of ORFs determining the synthesis of enzymes related to phage AP-16-3, was isolated from the rhizosphere of ginseng cultivated in the Republic of Korea [72]. The assumption of a wide geographic distribution of rhizobiophages similar to 16-3 is consistent with recently published data showing that *Rhizobium* phage 16-3 has been detected in sewage and lake water metaviromes in Northern Ireland [39,40].

Analysis of the pool of prophages related to phages AP-16-3 and 16-3 showed that these phages were actively involved in recombination processes. Thus, the frequency of occurrence of prophages that contained single ORFs related to phages AP-16-3 and 16-3 was eight times higher than that of prophages identified as being related to 16-3. It is likely that the genomes of temperate phages, as a result of repeated lysogenic cycles, lose the genes that enable their excision from the replicon of insertion, resulting in the appearance of incomplete prophages and questionable sequences. The flip side of this process is the fixation of new genetic sequences in the genome of the host bacterium, which may be significant for the ecological viability of the microorganism. The presence of a significant number of incomplete and questionable sequences of phage origin, as well as blocks and/or individual phage genes within unrelated phages, allows us to state the fact that the life cycle of a temperate phage, namely, the incorporation of its genome into the host bacterial genome, is not a stage of “conservation” of the phage with its subsequent release, as it has often been considered [73,74], but rather leads to its inactivation as a result of the loss of functionally significant genes and the gradual dissolution of the genome of the temperate phage in the host bacterium’s genome. This process plays an evolutionarily important role in the significant updating of the genetic pool of the host bacterium and may play a role in the leapfrog scenario of bacterial evolution.

Summarizing, *Sinorhizobium* phage AP-16-3 studied in this work and the prophage in strain *S. meliloti* NV1.1.1 containing the greatest number of ORFs homologous to *Rhizobium* phage 16-3, whose genome was studied in 1960s, were isolated by us in legume diversity foci in NW Dagestan and NW Kazakhstan (Figure S1), relating to the corresponding origins of cultivated plant diversity identified by Vavilov N.I. in the Caucasus and Central Asia, respectively, in contrast to the genomes of rhizobia strains of different geographical origins (strains from the laboratory collection and NCBI database), which overwhelmingly contained single ORFs homologous to phage 16-3. The presented data suggest that the gene centers of diversity of cultivated plants can also be used to study the diversity of microorganisms and viruses.

## 5. Conclusions

The results of our study are intended to fill the existing deficit of information on the geographical distribution and viability of temperate tailed rhizobiophages. The obtained evidence of phages related to 16-3 indicates the high transductive activity of temperate phages in relation to representatives of different species and genera of nitrogen-fixing nodule bacteria and, for the first time, allows us to discuss the specificity of phages in relation to a particular species/genus of nodule bacteria. It is shown that more than thirty phage sequences localized on chromosomes, megaplasmids, and cryptic plasmids can be present simultaneously in one rhizobia genome and can be related to one or many different phages belonging to different families, subfamilies, and classes. There is evidence of active participation of rhizobiophages in horizontal gene transfer, and data indicate their involvement in intragenomic recombination involving different phages (prophages), which leads to mosaicism of viral genomes. The screening performed allowed us to gain a new perspective on the evolutionary significance of the lysogenic cycle of temperate bacteriophages and suggested that the incorporation of phage genetic material into the host bacterial genome leads to the dissolution of phage genomes and their radical renewal and increases the potential ability of new phage genomes to appear. Joint analysis of rhizobiophages and prophages has significantly broadened our knowledge about the viability as well as the evolution of temperate phages.

## Figures and Tables

**Table 1:** Comparative characterization of *Sinorhizobium* phage AP-16-3 and *Rhizobium* phage 16-3.

Characteristics	*Sinorhizobium* Phage AP-16-3	*Rhizobium* Phage 16-3
Size, kbp	61.0	60.2
GC content, %	59.22	58.95
Number of ORFs	62	62 *
Common ORFs (nucleotide/amino acid)	42/45	

* Annotation based on eggNOG-mapper v2.

**Table 2:** List of PhRS-16-3-like prophages (according to PHASTER).

Species		Strain ^1^	Length, kb	GC, %	Completeness	Number of ORFs		
						Total	Phage Hit Proteins ^2^	Identical to *Rhizobium* Phage 16-3
*Sinorhizobium* sp.	*S. meliloti*	NV1.1.1	64.2	59.40	Int ^3^	105	71	51
		AK76	32	58.58	Quest ^4^	35	21	13
		CCMM B554 (FSM-MA)	36.3	57.99	int	36	22	12
		ABS7	36.3	57.96	int	40	23	12
	*S. medicae*	WSM419	32.1	59.12	int	44	29	14
	*S. fredii*	CCBAU 25509	41.8	58.14	quest	36	21	16
*Rh. lentis*		BLR27	21.5	60.40	quest	28	20	12
*Rh.* spp.		NLR16a	39.5	58.76	int	42	24	12
*Rh. leguminosarum*		OyaliB	63.7	58.69	int	100	63	41
*Mesorhizobium* spp.		DCY119	104.2	59.90	int	116	78	14

^1^ Strain BioSamples are listed in Table S2; ^2^ according to PHASTER; ^3^ intact phage; ^4^ questionable phage.

**Table 3:** Presence of ORFs belonging to the L region of AP-16-3 phage in PhRS-16-3-like prophages (PHASTER annotation).

ORF	Product	Prophages in Genomes									
		*S. meliloti*				*S. medicae* WSM419	*S. fredii* CCBAU 25509	*Rh lentis* BLR27	*Rh* sp. NLR16a	*Rh leguminosarum* OyaliB	*Mesorhizo bium* sp. DCY119
		NV1.1.1	AK76	CCMM B554 (FSM-MA)	ABS7						
p001 (terL)	terminase large subunit	-	-	-	-	-	-	+	+	+	-
p002 (terS)	terminase small subunit	-	-	-	-	-	-	+	+	+	-
p003	portal protein	-	-	-	-	-	+	+	+	+	-
p004	protease	-	-	-	-	-	+	+	+	+	-
p005a	major capsid protein	-	-	-	-	-	+	+	+	+	-
p007	hp *	-	-	-	-	-	+	+	+	-	+
p008	head–tail connector protein	-	-	-	-	+	+	+	+	+	+
p009	hp *	+	+	+	+	+	+	+	+	+	-
p010	head closure protein	+	-	+	-	-	+	-	-	+	-
p011	tail protein	+	+	+	+	+	+	-	-	+	-
p012	hp *	+	+	+	+	+	+	+	+	+	-
p013	hp *	+	-	-	-	-	-	-	-	-	-
p014	tail tube protein	+	+	+	+	+	+	-	-	+	-
p015	gene transfer agent family protein	+	+	-	-	+	+	-	-	+	-
p016	hp *	+	-	-	-	-	-	-	-	-	-
p017	tail length tape measure family protein	+	+	+	+	+	+	+	+	+	-
p018	tail protein	+	+	+	+	+	+	-	-	+	-
p019	hp *	+	+	+	+	+	+	-	-	-	-
p020	tail protein	+	+	+	+	+	+	-	-	+	-
p021	hp *	+	+	+	+	+	-	-	-	+	-
h	tail fiber protein H **	+	+	+	+	+	+	-	-	+	-
p023	kinase	+	+	+	+	+	-	-	-	+	-

ORFs encoding late viral proteins in PhRS-16-3-like prophages are marked in green; hp *, hypothetical protein; **, ORF encoding tail fiber protein H was represented in *S. meliloti* ABS7 strain by full-length and truncated sequences; the truncated sequence was not considered in the phylogenetic analysis.

## Data Availability

The assemblies and sequence data have been uploaded to the NCBI.

## Supplementary Materials

The following supporting information can be downloaded at: https://www.mdpi.com/article/10.3390/v15081701/s1, Figure S1: Intergenomic similarity values (right half) and alignment indicators (left half and top annotation) between phage phiLM21 and phiLM21-like prophages calculated using VIRIDIC; Table S1: *Sinorhizobium meliloti* lytic phages whose genome has been sequenced and analyzed; Table S2: Abundance of PhRS in genomes of *Sinorhizobium* spp., *Rhizobium* spp., and *Mesorhizobium* sp. strains (according to PHASTER); Table S3: Restriction sites in genomes of *Sinorhizobium* phage AP-16-3 и *Rhizobium* phage 16-3; Table S4: Open reading frames in the genome of *Sinorhizobium* phage AP-16-3 [75,76].
