# Supplementary material for: A Temperate Sinorhizobium Phage, AP-16-3, Closely Related to Phage 16-3: Mosaic Genome and Prophage Analysis
Source: Viruses. 2023 Aug 6;15(8):1701. doi: 10.3390/v15081701 (PMC10460002; doi:10.3390/v15081701)
Supplement: Supplementary file 1 [file viruses-15-01701-s001.zip › Fig Supl.pdf]

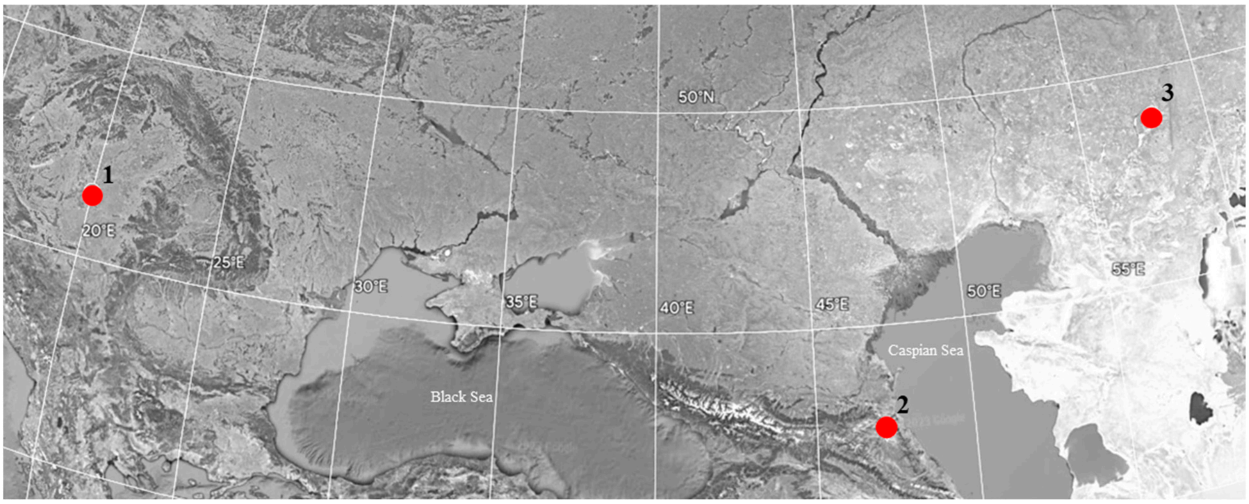

Figure S1. Map showing the soil sampling sites. Sampling sites were marked with points and studied bacteriophages:

1. *Rhizobium* phage 16-3, Hungary

2. *Sinorhizobium* phage AP-16-3, Dagestan, Russia

3. *S. meliloti* NV1.1.1, Mugodzhar, Kazakhstan

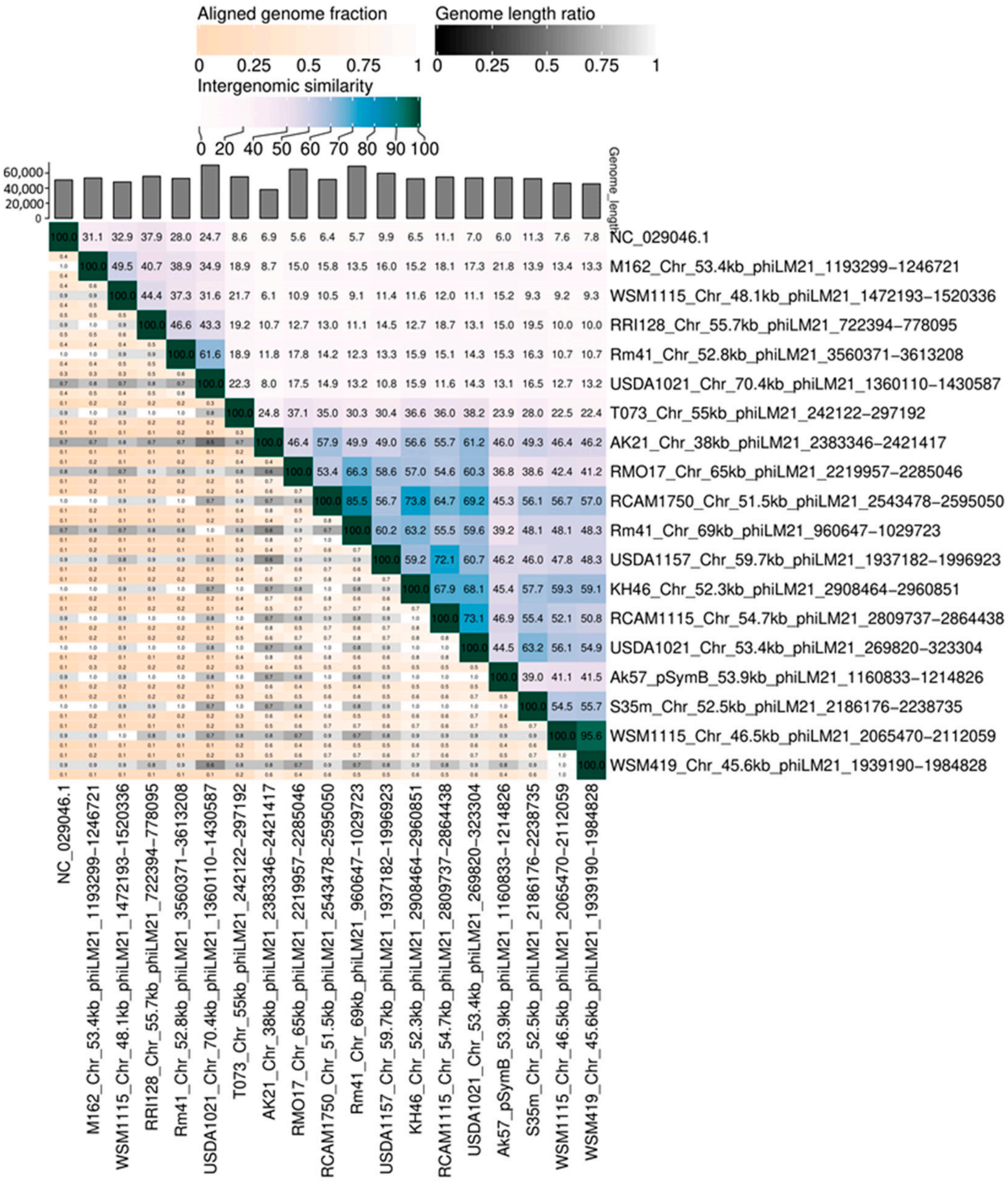

Figure S2. Intergenomic similarity values (right half) and alignment indicators (left half and top annotation) between phage phiLM21 and phiLM21-like prophages calculated using the VIRIDIC program [54].

In the right half, the numbers represent the similarity values for each pair of genomes. The left half presents three indicator values for each pair of genomes, in top-to-bottom order: aligned fraction genome 1 (for the genome found in this row), genome length ratio (for the two genomes in this pair) and aligned fraction genome 2 (for the genome found in this column).
