# Supplementary material for: A Temperate Sinorhizobium Phage, AP-16-3, Closely Related to Phage 16-3: Mosaic Genome and Prophage Analysis
Source: Viruses. 2023 Aug 6;15(8):1701. doi: 10.3390/v15081701 (PMC10460002; doi:10.3390/v15081701)
Supplement: Supplementary file 1 [file viruses-15-01701-s001.zip › Table S1. Sinorhizobium meliloti lytic phages whose genome has been sequenced and analyzed.pdf]

Table S1. *Sinorhizobium meliloti* lytic phages whose genome has been sequenced and analyzed

Phage	Country	Site	Source	RefSeq	Reference
phiM5	USA	-	-	MF074189.1	27
phiM6	USA	-	commercial alfalfa inoculants	MH700630	76
phiM7	-		soil from an alfalfa field	NC_041929.1	27
phiM9	-	-	commercial alfalfa inoculants	NC_028676.1	25
phiM12	USA	-	commercial alfalfa inoculants	NC_027204.1	27
phiM19	-	-	-	KR052481.1	75
phiN3	-	-	soil from an alfalfa field	NC_028945.1	75
P_ort11	Spain	Huelva	*M. marina* -rhizosphere sand samples	NC_049469.1	21
PBC5	Germany	Strassmoos field	soil	NC_003324.1	-
HMSP1-Susan	France	Salses	agricultural soil	MG214783.2	28
Rhizobium phage 16-3	Hungary	-	soil	NC_011103.1	30
phiLM21	Poland	Lubin copper mine	mineral sediments	NC_029046.1	29
