# Supplementary material for: A Temperate Sinorhizobium Phage, AP-16-3, Closely Related to Phage 16-3: Mosaic Genome and Prophage Analysis
Source: Viruses. 2023 Aug 6;15(8):1701. doi: 10.3390/v15081701 (PMC10460002; doi:10.3390/v15081701)
Supplement: Supplementary file 1 [file viruses-15-01701-s001.zip › Table S2. PhRS abundance in the genomes of Sinorhizobium spp., Rhizobium spp. and Mesorhizobium sp. strains (according to PHASTER).pdf]

Table S2. PhRS abundance in the genomes of *Sinorhizobium* spp., *Rhizobium* spp. and *Mesorhizobium* sp. strains (according to PHASTER)

Species	Strain	BioSample	Replicon (chromosome/plasmid)	Number of prophages		
				on replicon	PhRS-16-3-like***	PhRS-16-3-orph****
	Rm2011	SAMN02603522	chr	1	0	0
			pSymA	4	0	0
			pSymB	1	0	0
	Rm1021	SAMEA3283068	chr	1	0	0
			pSymA	4	0	0
			pSymB	2	0	0
	AK76*	SAMN17104055	chr	2	1[1/2]	0
			pSymA	3	0	0
			pSymB	1	0	0
			pAK76	3	0	0
	L6-AK89*	SAMN22420025	chr	2	0	0
			pCpl	3	0	0
			pSymA	0	0	0
			pSymB	3	0	0
	AK83*ɸ	SAMN00017059	chr 1	8	0	0
			chr 2	2	0	0
			chr 3	7	0	0
			pSINME01	3	0	0
			pSINME02	0	0	0
	AK21*ɸ	SAMN08428886	chr	9	0	2
			pSymA	2	0	0
			pSymB	2	0	0
			pSmeAK21a	2	0	0
			pSmeAK21b	2	0	0
	AK57*ɸ	SAMN24288876	chr	4	0	2
			pSymA	6	0	0
			pSymB	3	0	0
	S35m**ɸ	SAMN16812329	chr	2	0	1
			pSymA	2	0	0
			pSymB	1	0	0
	NV1.1.1.**	this work	chr	2	1[1/2]	0
			pSymA	0	0	0
			pSymB	2	0	0
	B401	SAMN06227501	chr	1	0	0
			pSymA	1	0	0
			pSymB	3	0	0
	USDA1021ɸ	SAMN07175167	chr	5	0	2[2]
			accessoryA	5	0	0
			psymA	2	0	0
			psymB	2	0	1
	USDA1157ɸ	SAMN07175169	chr	8	0	1
			accessoryA	0	0	0
			psymA	2	0	0

Sinorhizobium meliloti			psymB	2	0	1[1]
	USDA1106	SAMN07175168	chr	2	0	0
			psymA	2	0	0
			psymB	2	0	0
	RU11/001[Φ]	SAMEA3146337	chr	8	0	0
			pSymA	5	0	0
			pSymB	2	0	0
			pSmeRU11a	0	0	0
			pSmeRU11b	1	0	0
			pSmeRU11c	1	0	0
			pSmeRU11d	3	0	0
	SM11[Φ]	SAMN02603056	chr	7	0	0
			pSmeSM11c	7	0	0
			pSmeSM11d	3	0	0
			pSmeSM11a	0	0	0
			pSmeSM11b	0	0	0
	BL225C	SAMN00017103	chr	4	0	0
			pSINMEB01	0	0	0
			pSINMEB02	3	0	0
	GR4	SAMN02603224	chr	2	0	0
			pRmeGR4a	0	0	0
			pRmeGR4b	3	0	0
			pRmeGR4c	1	0	0
			pRmeGR4d	2	0	0
	B399[Φ]	SAMN06229775	chr	2	0	0
			pSym	4	0	0
			pSymA	1	0	0
	RRI128[Φ]	SAMN23416898	chr	3	0	1[2]
			pRRI128_1	3	0	0
			pRRI128_2	1	0	0
			pRRI128_3	5	0	0
			pRRI128_4	3	0	0
			pRRI128_5	2	0	0
	KH46[Φ]	SAMN07175162	chr	2	0	1[2]
			psymA	2	0	0
			psymB	3	0	0
	KH35c[Φ]	SAMN07175161	chr	3	0	0
			accessoryA	0	0	0
			psymA	1	0	0
			psymB	4	0	0
	HM006[Φ]	SAMN07175160	chr	4	0	2[2]
			accessoryA	0	0	0
			psymA	4	0	0
			psymB	3	0	0
	RMO17[Φ]	SAMN02952139	chr	2	1[4]	1[2]
			pSymA	1	0	0
			pSymB	2	0	0
	CCMM B554 (FSM-MA)	SAMN06284128	chr	3	1[1/2]	1
			pSymA	3	0	0
			pSymB	1	0	0

	Rm41[3]ϕ	SAMN07175165	chr	4	0	2[2]
			accessoryA	1	0	0
			psymA	3	0	0
			psymB	3	0	0
	RCAM1115ϕ	SAMN14403975	chr	13	0	1
			p_1	4	0	0
			p_2	3	0	0
			p_3	3	0	0
	RCAM1750ϕ	SAMN14403976	chr	3	0	1[2]
			p_1	5	0	0
			p_2	1	0	0
	ABS7	SAMN18865712	chr	4	1[1/2]	1
			pSymA	2	0	0
			pSymB	1	0	0
	M270ϕ	SAMN07175164	chr	9	0	3
			accessoryA	6	0	0
			accessoryB	4	0	0
			accessoryC	1	0	0
			psymA	9	0	0
			psymB	1	0	0
	M162ϕ	SAMN07175163	chr	6	0	2[2]
			accessoryA	2	0	0
			psymA	2	0	0
			psymB	1	0	0
	T073ϕ	SAMN07175166	chr	5	0	1[2]
			accessoryA	0	0	0
			psymA	2	0	0
			psymB	1	0	0
**In total for *S. meliloti***	**31**		**121**	**339**	**5[4]**	**27**
					**31**	
S. medicae	WSM419ϕ	SAMN02598363	chr	4	1[1/2]	2[2]
			pSMED01	1	0	0
			pSMED02	5	0	0
			pSMED03	3	0	0
	WSM1115ϕ	SAMN23416899	chr	13	0	2[2]
			pWSM1115_1	1	0	0
			pWSM1115_2	4	0	0
			pWSM1115_3	2	0	0
S. fredii	CCBAU 25509	SAMN03761947	chr	5	1[1/2]	0
			pSF25509a	4	0	0
			pSF25509b	1	0	0
	NGR234	SAMN02603283	chr	4	0	0
			pNGR234a	2	0	0
			pNGR234b	2	0	0
	CCBAU 83666ϕ	SAMN07445561	chr	3	0	1
			pSF83666a	7	0	0
			pSF83666b	4	0	0
			pSF83666d	1	0	1
	NXT3ϕ	SAMN07824032	chr	1	0	0
			pSfreNXT3a	2	0	0

	NXT3[ϕ]	SAMN07824032	pSfreNXT3b	2	0	0
			pSfreNXT3c	0	0	0
	CCBAU 45436	SAMN03761948	chr	3	0	0
			pSF45436a	5	0	0
			pSF45436b	1	0	0
			pSF45436d	2	0	0
			pSF45436e	1	0	0
**In total for *Sinorhizobium* spp.:**	**38**		**168**	**422**	**7[4]**	**33**
					**39**	
Rh. lentis	BLR27	SAMN18139228	chr	3	1	0
			p1	0	0	0
			p2	0	0	0
			p3	0	0	0
			p4	1	0	0
Rh spp.	NLR16a	SAMN18525573	chr	2	1	0
			p1	0	0	0
			p2	0	0	0
			p3	0	0	0
			p4	1	0	0
			p5	0	0	0
			p6	0	0	0
			p7	1	0	0
Rh. leguminosarum	OyaliB	SAMN18139820	chr	1	1[1]	0
			p1	0	0	0
			p2	0	0	0
			p3	2	0	0
			p4	0	0	0
			p5	0	0	0
Mesorhizobium sp.	DCY119	SAMN09829652	chr	4	1	1[5]
**In total:**	**42**		**188**	**437**	**11[4]**	**34[5]**
					**43**	

[1]- prophage contains an ORF encoding a full-length TfpH protein (in the case of *S. meliloti* ABS7 strain carrying a full-length and a truncated sequence, the truncated sequence is not involved in the phylogenetic analysis); [2] – prophage contains an ORF encoding p019 protein (in the case of *S. meliloti* USDA1021, HM006 and M162 and *S. medicae* WSM419 strains, on one prophage; in the case of *S. meliloti* Rm41 and *S. medicae* WSM1115 strains, on both prophages); [3] – BioSample SAMN07175165 analyzed; [4] – prophage of *S. meliloti* RMO17 with low nucleotide similarity according to BLASTn (Cov < 1%), was not analyze; [5] – PhRS-orph-16-3 of *Mesorhizobium* sp. DCY119 did not analyze; [*] – strains isolated from saline Mugodzhar region, part of the introgressive center of alfalfa hybridization in NW Kazakhstan; [**] – strains isolated from mountainous region of Dagestan, part of the origin of diversity of cultivated plants in the Caucasus; *** - prophages similar to phage 16-3 according to Phaster; **** - prophages contained single ORFs related to phage 16-3; [ϕ] – genomes contained phiLM21-like prophages
