# Supplementary material for: A Temperate Sinorhizobium Phage, AP-16-3, Closely Related to Phage 16-3: Mosaic Genome and Prophage Analysis
Source: Viruses. 2023 Aug 6;15(8):1701. doi: 10.3390/v15081701 (PMC10460002; doi:10.3390/v15081701)
Supplement: Supplementary file 1 [file viruses-15-01701-s001.zip › Table S3. Restriction sites in Sinorhizobium phage AP-16-3 and Rhizobium phage 16-3 genomes.pdf]

Table S3. Restriction sites in *Sinorhizobium* phage AP-16-3 and *Rhizobium* phage 16-3 genomes

Endonuclease	Endonuclease restriction site	Number of restriction sites (in silico) in phage genome:	
		Sinorhizobium phage AP-16-3	*Rhizobium* phage 16-3
Eco RI	GAATTC	25	16
Hind III	AAGCTT	13	11
Gsu I	CTGGAG	25	21
Bsu RI	GGCC	387	397
Alu I	AGCT	180	164
Bam HI	GGATCC	17	20
Hpa II	CCGG	298	316
Hinf I	GANTC	119	121
Hha I	GCGC	767	742
Hae II	RGCGCY	159	154
Taq I	TCGA	437	419
